# Assessment of novel inflammatory biomarkers in patients with peripheral vertigo

**DOI:** 10.1097/MD.0000000000044573

**Published:** 2025-09-19

**Authors:** Yeşim Yüksel, Cihan Bedel, Muhammet Yildiz, Fatih Selvi, Ökkeş Zortuk, Erdem Atalay Çetinkaya

**Affiliations:** a Department of Otorhinolaryngology, University of Health Sciences, Antalya Training and Research Hospital, Antalya, Turkey; b Department of of Emergency Medicine, University of Health Sciences, Antalya Training and Research Hospital, Antalya, Turkey; c Department of Emergency Medicine, Hatay Defne State Hospital, Hatay, Turkey.

**Keywords:** biomarker, inflammation, pan-immunoinflammatory index, systemic immune-inflammation index, systemic inflammatory response index, vertigo

## Abstract

Numerous studies have evaluated the relationship between peripheral vertigo (PV) and inflammation. In these studies, various biomarkers have been used as indicators of inflammation. Contradictory results have been reported regarding the presence of inflammation based on these previously assessed biomarkers in PV. The systemic inflammatory response index (SIRI) and systemic immune-inflammation index (SII), which indicate these inflammatory processes, as well as the pan-immune-inflammatory index (PIV), have not been clearly defined in PV. This study aims to investigate the relationship between the inflammatory biomarkers SIRI, SII, and PIV and PV.

This retrospective case-control study analyzed patients who were diagnosed with acute PV at a tertiary education and research emergency department. The study recorded demographic data of patients and values of white blood cells, platelets, neutrophils, lymphocytes, monocytes, eosinophils, basophils, and immature granulocytes obtained during emergency admission from these blood samples, SIRI, SII, and PIV values were calculated and compared between the control group.

A total of 1232 patients meeting the inclusion criteria were included in this study. Moreover, the study included 968 control subjects who shared similar age and demographic characteristics. SIRI, SII, and PIV values of the patients were significantly higher in vertigo patients than in the control group (1.50 vs 0.77, *P* <.001), (622.41 vs 393.47, *P* <.001), and (393.59 vs 184.21, *P* <.001), respectively. receiver operating characteristic curves were used to compare the diagnostic effectiveness of SIRI, SII, and PIV parameters to the control group. The optimal value for SIRI was found to have an area under the curve (AUC) of 0.760, sensitivity of 82.3, and specificity of 60.3 (*P* <.001).

PV can be caused by many diseases with various pathophysiological changes. There may be various mechanisms besides inflammation and atherosclerosis of microvascular structures. The study investigated the relationship between PV and novel systemic inflammatory biomarkers in vertigo patients, yielding clinically significant results. The findings revealed that SIRI, SII, and PIV were elevated in patients with PV and may serve as complementary diagnostic markers.

## 1. Introduction

Peripheral vertigo (PV), originating from the peripheral components of the vestibular system, can be associated with inflammatory processes in different parts of the body, including the inner ear, vascular system, and even systemic inflammatory diseases. Inflammation of peripheral vestibular structures due to viral infections, autoimmune reactions, or vascular pathologies can lead to vestibular dysfunction.^[1]^ Benign paroxysmal positional vertigo (BPPV) is the most common cause of PV, accounting for over half of all cases. Approximately 50% to 70% of BPPV cases occur with no known cause and are referred to as primary or idiopathic BPPV. The remaining cases are called secondary BPPV and are often associated with an underlying pathology, such as head trauma, vestibular neuronitis, labyrinthitis, Ménière disease, migraine, ischemia, and iatrogenic causes. Viral labyrinthitis or vestibular neuronitis accounts for up to 15% of BPPV cases.^[2–4]^ Many studies have shown that systemic or local inflammation can lead to or accompany peripheral vestibular vertigo.^[1,2,5–13]^

Previous studies have investigated inflammatory biomarkers such as the neutrophil-to-lymphocyte ratio (NLR) and platelet-to-lymphocyte ratio (PLR) to evaluate the relationship between PV and inflammatory processes.^[5–13]^ In recent years, there has been a growing body of research on the role of markers obtained by comparing parameters, such as systemic inflammatory response index (SIRI), and systemic immune-inflammation index (SII), pan-immunoinflammatory index (PIV) in various inflammation-related diseases.^[14–18]^ Although many studies have evaluated the relationship between inflammation and PV, novel inflammatory biomarkers such as PIV, SIRI, and SII have not been previously investigated. Therefore, our study aims to examine the relationship between parameters such as PIV, SIRI, and SII and PV.

## 2. Methods

This retrospective case–control study evaluates patients diagnosed with acute PV who presented to a tertiary hospital emergency department with dizziness complaints between January 2023 and January 2025. The study was conducted by the principles of the Helsinki Declaration and was approved by the local ethics committee.

**Figure 1.**
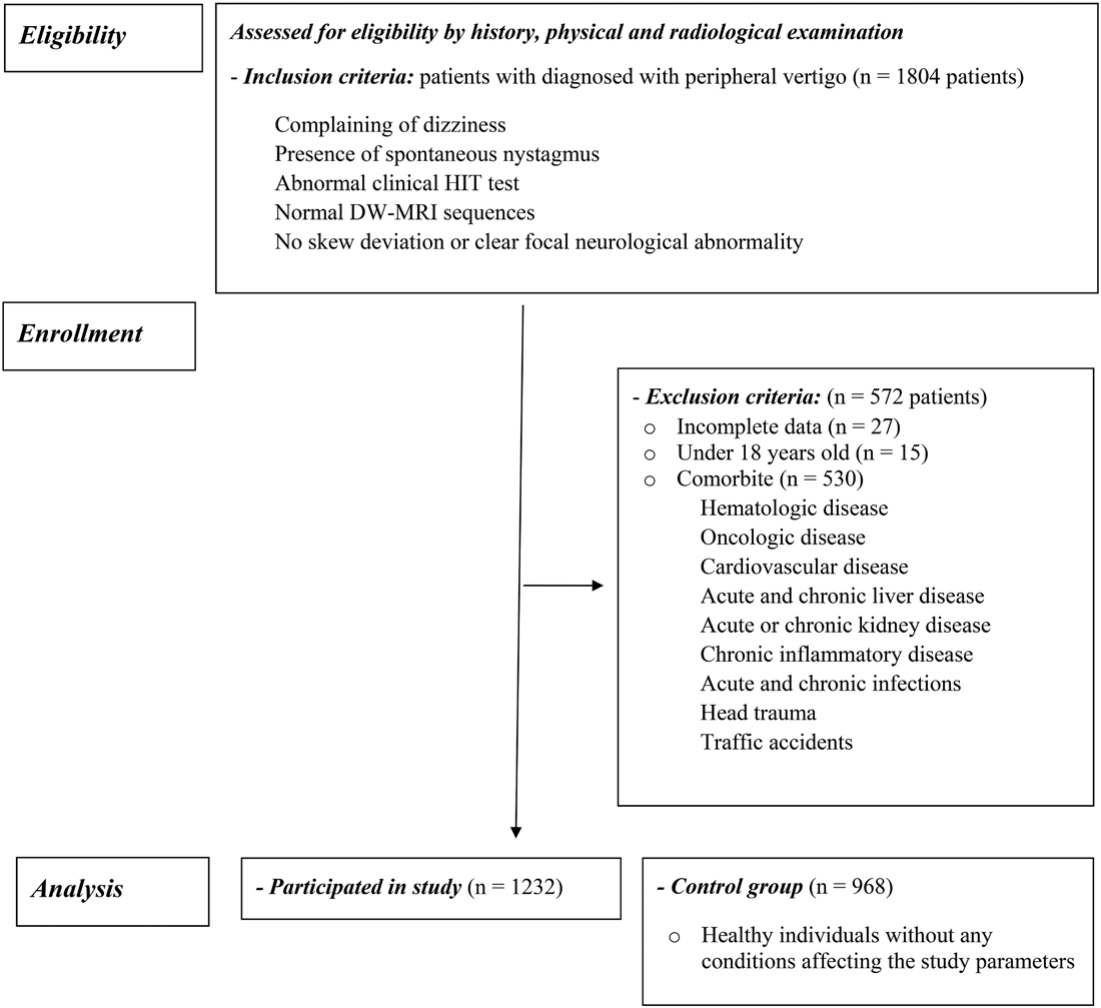
Study protocol: patient flow of PV cases throughout the study. PV = peripheral vertigo.

All patients underwent detailed neurological, otological, and cardiovascular examinations by a general practitioner in the emergency department. Assessments included complete blood count, basic biochemical tests, electrocardiography, and cranial imaging. Patients with a preliminary diagnosis of PV were further evaluated by an otolaryngologist for definitive diagnosis. Diagnosis of PV required the following criteria: presence of spontaneous nystagmus, abnormal clinical head impulse test, normal diffusion-weighted magnetic resonance imaging (DW-MRI) sequences, and no skew deviation or clear focal neurological abnormality. Patients with incomplete data and those under 18 years old were excluded from the study. Additionally, patients with hematologic, oncologic, cardiovascular, or central nervous system diseases, acute and chronic liver or kidney disease, diagnosed chronic inflammatory disease, acute and chronic infections, or a history of head trauma or traffic accidents were excluded. All patients aged 18 and above who received a definitive diagnosis of acute PV and met the inclusion criteria were included (Fig. 1).

Demographic data and laboratory values, including white blood cells (WBC), neutrophils, platelets, lymphocytes, monocytes, eosinophils, and hemoglobin values, were recorded at the time of emergency department admission. Blood samples obtained from patients were analyzed within the first 30 minutes. NLR was calculated as the simple ratio of absolute neutrophil count to lymphocyte count. PIV value, a new inflammation marker, was obtained by multiplying the neutrophil count by the platelet and monocyte counts and dividing this product by the lymphocyte count. SIRI value was calculated as the product of neutrophil and monocyte counts divided by the lymphocyte count. SII value was obtained by multiplying platelet and neutrophil counts and dividing the result by the lymphocyte count.

The control group consisted of healthy individuals without any conditions affecting the study parameters, selected from patients who visited the medical board of our hospital with similar age and gender characteristics. Healthy individuals also did not have a history of neurological abnormalities, no hearing problems or dizziness complaints, did not use any medications, and had normal neuro-otological physical examination. Groups were compared based on these parameters.

### 2.1. Statistical analysis

Data analysis was performed using SPSS 27.0 (SPSS Inc., Chicago). The Kolmogorov–Smirnov test was applied to assess the distribution of continuous variables. Mean ± standard deviation was used for continuous variables when comparing parameters and demographic data of patients diagnosed with vertigo. Frequencies and percentages (%) were used for categorical data. The Student *t* test was used to compare inflammatory parameters for variables showing normal distribution, while the Mann–Whitney *U* test was used for those that did not exhibit normal distribution. Youden index used to select the optimal predicted probability cutoff value. The diagnostic efficacy of the predictive model was gauged through the receiver operating characteristic (ROC) curve, with the area under the ROC curve (AUC) serving as a quantitative measure. A *P* <.05 was considered statistically significant.

## 3. Results

The study included 1232 patients who met the inclusion criteria. Additionally, 968 control subjects with similar age and demographic characteristics were enrolled. The mean age of patients in the vertigo group was 49.17 ± 18.75, while in the control group, it was 50.05 ± 17.47. Both groups were predominantly female. The vertigo group showed significantly higher levels of WBC, neutrophils, platelets, monocytes, basophil count, and immature granulocytes compared to the control group. In addition, lymphocyte counts were significantly higher in the control group compared to the vertigo group. Table 1 compares the demographic data and laboratory values of the 2 groups.

**Table 1:** Demographic characteristics of patients and control group.

	Control (n = 968)	Vertigo (n = 1232)	*P*-value
Female	552 (57.02%)	734 (59.58%)	.077
Age	50.05 ± 17.47	49.17 ± 18.75	.185
White blood cell (×10^3^/mm^3^) (mean ± SD)	7.76 ± 5.01	9.06 ± 3.12	**<.001**
Hemoglobin (g/dL) (mean ± SD)	13.07 ± 2.01	12.93 ± 2.08	.059
Platelet (×10^3^/mm^3^) (mean ± SD)	229.84 ± 101.54	272.31 ± 86.05	**<.001**
Neutrophil (×10^3^/mm^3^) (mean ± SD)	4.3 ± 2.42	6.03 ± 2.88	**<.001**
Lymphocyte (×10^3^/mm^3^) (mean ± SD)	2.36 (1.33)	2.11 (1.14)	**<.001**
Monocyte (×10^3^/mm^3^) (median. IQR)	0.57 ± 0.43	0.65 ± 0.25	**<.001**
Eosinophil (×10^3^/mm^3^) (median. IQR)	0.12 (0.15)	0.12 (0.14)	.278
Basophil (×10^3^/mm^3^) (mean ± SD)	0.042 ± 0.024	0.048 ± 0.026	**<.001**
Immature granulocyte (median. IQR)	0.02 (0.02)	0.04 (0.03)	**<.001**

Bold values indicate statistical significance (*P* < 0.05).

IQR = interquartile range, SD = standard deviation.

**Figure 2.**
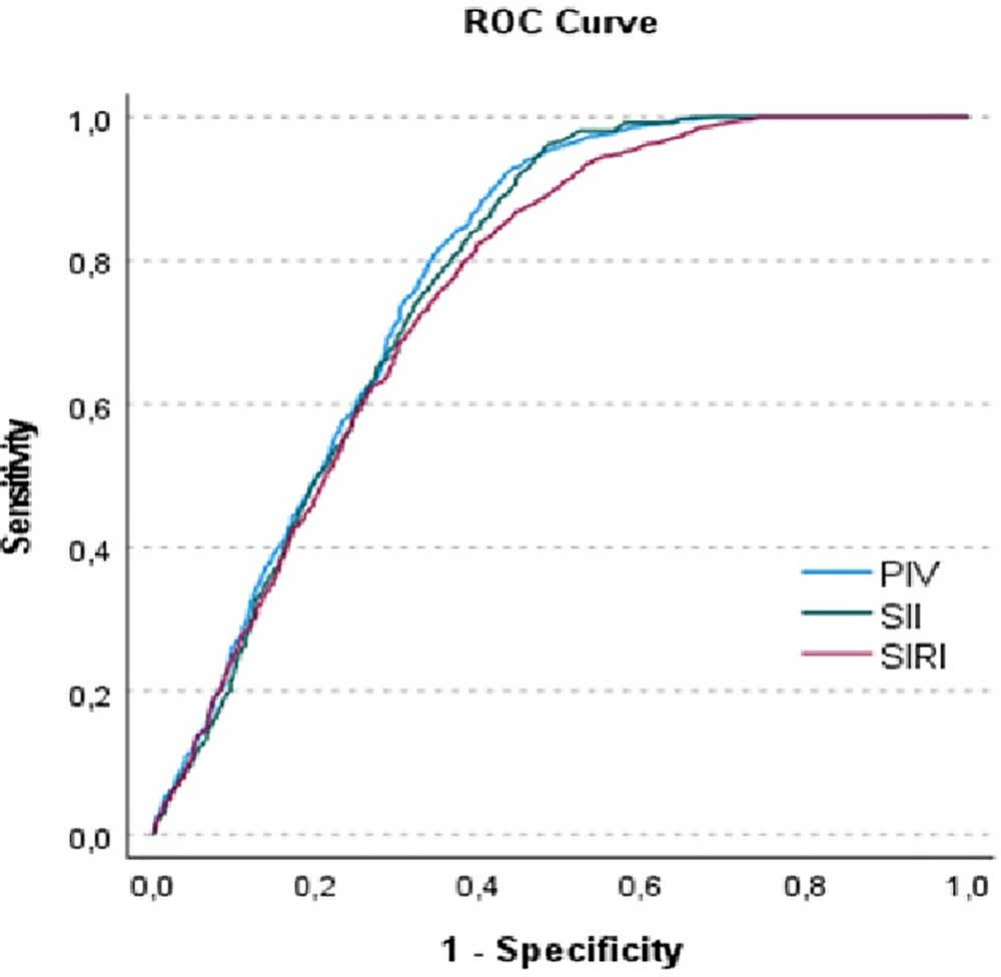
Receiver operating characteristic curves of inflammatory biomarkers to predict vertigo attack.

SIRI value of the patients was significantly higher in vertigo patients than in the control group (1.50 [1.38] vs 0.77 [0.78], *P* <.001). SII value of the patients was significantly higher in vertigo patients than in the control group (622.41 [555.78] vs 393.47 [483.18], *P* <.001), PIV value of the patients was also significantly higher in vertigo patients than in the control group (393.59 [413.53] vs 184.21 [275.83], *P* <.001). Table 2 compares laboratory values of the 2 groups. ROC curves were plotted to determine the effectiveness of these parameters in distinguishing the diagnosis, with an AUC of 0.760 for SIRI at the optimal value. The sensitivity was 82.3, and the specificity was 60.3 (*P* <.001), as shown in Table 3 and Figure 2.

**Table 2:** Comparison of the inflammatory biomarkers for the groups.

	Control (n = 968)	Vertigo (n = 1232)	*P*-value
SIRI (median. IQR)	0.77 (0.78)	1.50 (1.38)	**<.001**
SII (median. IQR)	393.47 (483.18)	622.41 (555.78)	**<.001**
PIV (median. IQR)	184.21 (275.83)	393.59 (413.53)	**<.001**
NLR (median. IQR)	2.09 (1.12)	2.28 (1.42)	.06

Bold values indicate statistical significance (*P* < 0.05).

IQR = interquartile range, NLR = neutrophil-to-lymphocyte ratio, PIV = pan-immunoinflammatory index, SII **=** systemic immuno-inflammation index, SIRI **=** systemic inflammatory response index.

**Table 3:** The receiver operating characteristic curves for inflammatory biomarkers.

	AUC	SD	*P*-value	Cut-off	Sensitivity	Specificity
SII	0.775	0.01	.001	308.94	69.1	51.8
SIRI	0.760	0.1	.001	0.9225	82.3	60.3
PIV	0.782	0.1	.001	180.1	89	7.7

AUC = area under the curve, PIV **=** pan-immunoinflammatory index, SD = standard deviation, SII = systemic immuno-inflammation index, SIRI **=** systemic inflammatory response index.

## 4. Discussion

Systemic inflammation biomarkers are cost-effective markers used in the diagnosis and prognosis of oncological, cardiovascular, and inflammatory diseases, including vertigo.^[5–19]^ The association between PV and vestibular neuritis and inflammation has been evaluated in various studies using markers such as CRP, WBC, NLR, PLR, and mean platelet volume (MPV).^[5–13]^ Although studies have been conducted using biomarkers such as SII, SIRI, and PIV in oncological, cardiovascular, and inflammatory diseases, there is no study has evaluated the relationship between PV and inflammation using these novel biomarkers.^[14–19]^

In their study involving 103 patients, Özbay et al investigated NLR as an inflammatory marker in patients with PV and found it to be significantly higher than in healthy individuals. Therefore, they reported that NLR is a new marker that should be considered in the evaluation of patients with PV.^[5]^ Similarly, while some studies have reported that multiple different inflammatory biomarkers are effective indicators in patients with PV,^[9–13]^ other studies have reported the opposite.^[6–8]^ Temirbekov and colleagues reported that, unlike the findings of Özbay and colleagues, there was no statistically significant difference between the patient and control groups in terms of NLR, PLR, or MPV. However, although the number of patients in their study was similar to that in Özbay et al.’s study, they noted that this could be due to the small sample size.^[6]^ In our study, although the NLR values of our PV patients were higher than those of the control group, no statistically significant difference was found.

NLR, PLR, and MPV are repeatable, inexpensive, and widely available parameters that serve as markers of inflammation and immune response. They are even considered to be as valuable as more expensive inflammatory markers such as IL-6, IL-1β, and tumor necrosis factor-α.^[6]^ The reporting of conflicting results in different studies regarding various inflammatory parameters should not be a reason for debate regarding the association of inflammatory processes in the etiopathogenesis of PV patients. The use of cost-effective biomarkers in these studies is a reasonable approach. However, the efficacy and/or patient numbers of other biomarkers evaluated in previous studies, aside from SII, SIRI, and PIV, which were assessed in our study, may have been insufficient.

In the last 50 years, cupulolithiasis–canalolithiasis (the mechanical theory) has become the accepted theory, however, we are still unable to clarify the physiopathology of PV fully. There are numerous studies demonstrating the association of PV with various pathologies such as autoinflammatory and autoimmune causes, inflammatory processes caused by emotional state changes, vasculitis that may arise from microvascular inflammation, atherosclerosis, and atherothrombosis.^[1,2,5–7,10,11,20]^

Both autoimmune and autoinflammatory diseases can affect the inner ear, causing vestibular dysfunction and vertigo.^[1]^ As stated in the study by Athanasopoulos et al., autoinflammatory diseases are characterized by uncontrolled inflammatory attacks, and this inflammation stems from dysregulated responses of the innate immune system, particularly involving components such as cytokines and inflammasomes.^[1]^ In primary or idiopathic BPPV, which is cited as the most important cause of PV, studies have reported that inflammation is the underlying condition leading to vestibulopathy. One of the most important indicators of inflammation in these PV patients is the effective clinical response to steroids, a potent anti-inflammatory agent, in cases of maneuver therapy-resistant BPPV.^[2]^

There are studies in the literature evaluating the association between anxiety and depression in patients with PV.^[21,22]^ These studies have reported emotional changes associated with vertigo. Despite this, it has not been reported that inflammation caused by stress originating from an emotional state may also cause vertigo. Hormonal changes associated with psychological and physiological stress, activation of the sympathetic nervous system, and increased levels of proinflammatory cytokines, which are also elevated in oxidative stress, further indicate inflammation.^[5,20]^ In previous studies evaluating inflammatory biomarkers in patients with PV, inflammation associated with anxiety and stress, or microvascular inflammation caused by the inflammatory disease process in the patient were cited as reasons. These studies reported that vestibular disorders develop due to atherosclerosis and atherothrombosis caused by microvascular inflammation, and that vasospasm and atherosclerosis also predispose to calcium crystal accumulation in the semicircular canals.^[5–7]^

Inflammatory biomarkers have advantages over changes in WBC, such as neutrophils, monocytes, and lymphocytes, as well as platelets. Biomarkers indicating inflammatory, immunological, and thromboembolic processes can remain more stable, being less affected by physiological and pathological conditions.^[10,11]^ More complex novel biomarkers, such as SIRI, SII, and PIV, demonstrate superior ability to detect changes in blood cells during inflammation compared to biomarkers like NLR and PLR, due to their formulation. The large sample size in our study, the increase in SIRI, SII, and PIV, inflammatory biomarkers, was found to be much more significant than the increase in NLR, which was insufficient in demonstrating the association between PV and inflammation.

This study has some limitations. First, our study has a retrospective and single-centered design. In this study, which aimed to evaluate the relationship between vertigo and inflammation, the inability to perform time-dependent dynamic laboratory monitoring is one of the most important limitations. Considering that inflammation biomarkers such as PIV, SIRI, and SII may require dynamic monitoring, it should be acknowledged that relying solely on values obtained at the time of presentation and during hospitalization limits the analytical power of this study. Therefore, further prospective study designs should be conducted to investigate these new biomarkers in subgroups of PV causes such as primary BPPV, Meniere disease, vestibular neuritis, labyrinthitis, superior canal dehiscence syndrome, and perilymphatic fistula; taking into account different age groups and gender differences, will enhance the clinical significance of the findings.

PV is not a primary disease but a symptom that can be triggered by various diseases with different pathophysiologies. Our study is the first in the literature to investigate the relationship between PV and new systemic inflammatory indices (SIRI, SII, PIV). We believe that SIRI, SII, and PIV could be evaluated as new biomarkers in the diagnosis and follow-up of patients with PV accompanied by different inflammatory processes, based on current knowledge regarding their roles in inflammatory and autoimmune processes.

## Author contributions

**Conceptualization:** Yeşim Yüksel, Cihan Bedel.

**Data curation:** Yeşim Yüksel, Cihan Bedel, Fatih Selvi, Ökkeş Zortuk.

**Formal analysis:** Yeşim Yüksel, Muhammet Yildiz, Fatih Selvi, Ökkeş Zortuk.

**Investigation:** Yeşim Yüksel, Cihan Bedel, Fatih Selvi, Ökkeş Zortuk.

**Methodology:** Yeşim Yüksel, Cihan Bedel, Muhammet Yildiz, Fatih Selvi, Ökkeş Zortuk, Erdem Atalay Çetinkaya.

**Project administration:** Yeşim Yüksel, Cihan Bedel, Erdem Atalay Çetinkaya.

**Resources:** Yeşim Yüksel, Muhammet Yildiz, Erdem Atalay Çetinkaya.

**Software:** Yeşim Yüksel, Cihan Bedel, Muhammet Yildiz, Ökkeş Zortuk.

**Supervision:** Yeşim Yüksel, Muhammet Yildiz, Fatih Selvi, Erdem Atalay Çetinkaya.

**Validation:** Yeşim Yüksel, Cihan Bedel, Fatih Selvi, Ökkeş Zortuk.

**Visualization:** Yeşim Yüksel.

**Writing – original draft:** Yeşim Yüksel, Cihan Bedel, Muhammet Yildiz, Ökkeş Zortuk.

**Writing – review & editing:** Yeşim Yüksel, Cihan Bedel, Muhammet Yildiz, Fatih Selvi, Erdem Atalay Çetinkaya.
